# Critical importance of patient-reported outcomes for a comprehensive assessment of psoriatic arthritis patients

**DOI:** 10.3389/fmed.2023.1332432

**Published:** 2024-01-16

**Authors:** Marika Lo Monaco, Raffaella Mallaci Bocchio, Giuseppe Natoli, Annarita Giardina, Ignazio Cangemi, Salvatore Scibetta, Christiano Argano, Salvatore Corrao

**Affiliations:** ^1^Department of Health Promotion Sciences, Maternal and Infant Care, Internal Medicine and Medical Specialties [PROMISE], University of Palermo, Palermo, Italy; ^2^Department of Clinical Medicine, Internal Medicine Unit with Rheumatology, Dermatology, Diabetology and Tertiary Diabetic Foot Healthcare, National Relevance and High Specialization Hospital Trust ARNAS Civico, Di Cristina, Benfratelli, Palermo, Italy

**Keywords:** patient reported outcomes, nurse assessment, nursing, psoriatic disease, disease activity, quality of life, rheumatology, multidimensional assessment

## Abstract

**Introduction:**

Psoriatic arthritis (PsA) is a heterogeneous, chronic inflammatory disease that negatively impacts patients’ quality of life. Patient-reported outcome measures (PROMs) are used to capture patient perspectives in disease assessment, and physicians use the Disease Activity Index for Psoriatic Arthritis (DAPSA) to evaluate disease activity in PsA. The study aimed to assess the relationship between PROMs and the DAPSA score in consecutive outpatients affected by PsA.

**Materials and methods:**

A cross-sectional study was conducted from March 2018 to October 2020 at the PsA clinic of the ARNAS Civico in Palermo (Italy), enrolling outpatients with PsA. Patients were assessed for their disease activity according to the DAPSA score, and PROMs, such as PHQ-9, HAQ, FACIT-F, and PsAID, were evaluated. Linear regression analysis evaluated the relationship between the DAPSA Score and the included PROMs.

**Results:**

158 PsA consecutive peripheral subset psoriatic arthritis outpatients were recruited. The median years of illness was 10.6 (9.3–11.9), and the median DAPSA score was 19.02 (9–33.1). The regression analysis highlighted a strong relationship between the DAPSA score and the PsAID (adjR^2^ 26%, *p* < 0.0001), the FACIT-F (adjR^2^ 25.4%, p < 0.0001), the HAQ (adjR^2^ 23.7%, *p* < 0.0001), and PHQ-9 (adjR^2^ 15%, *p* < 0.0001).

**Conclusion:**

PROMs are strongly associated with the DAPSA score, but it allows in-depth evaluation of the impact of the disease on different domains of PsA patients’ life.

## Introduction

Psoriatic Arthritis (PsA) is a chronic inflammatory arthritis associated with psoriasis (1). It has been estimated prevalence of 0.1 to 1% in the general population around the world, and a pooled prevalence of 19.7% (95% CI 18.5–20.9%) in patients with psoriasis (2). PsA involves either joints or cutaneous mantle, and the Classification of Psoriatic Arthritis (CASPAR) criteria (3) is a helpful diagnostic tool to screen the population. According to these criteria, the patient must show signs of inflammatory articular disease (joint, spine, or enthesis) with at least 3 points out of five categories as psoriasis (current, or personal or family history), psoriatic nail dystrophy, absence of rheumatoid factor, dactylitis (current or personal history), and radiological evidence of new juxta-articular bone formation. PsA affects multiple organ systems, including peripheral and axial joints, entheses, skin, and nails, and it is associated with comorbidities such as osteoporosis, uveitis, overt bowel inflammation, cardiovascular disease, and depression/mental health disorders (4). Therapy in PsA aims to control the inflammation process, slowing and preventing structural damage and complications. Different drugs are used, such as non-steroidal anti-inflammatory drugs (NSAIDs), synthetic disease-modifying anti-rheumatic drugs (DMARDs), biologic therapies, or novel small molecules.

Physicians evaluate therapy effectiveness by assessing patients with PsA through specific clinical tools such as the Disease Activity Index for Psoriatic Arthritis (DAPSA) score (5). This instrument is based on the summation of five variables: tender and swollen joints (TJC68, SJC66), patient’s global assessment (PtGA) and Patient Pain on a 10 cm visual analog scale (VAS), as well as C-reactive protein (CRP). PsA has a significant impact on a patient’s physical function, social participation, mood, and quality of life. For these reasons, patient experience plays a central role in a more comprehensive assessment of rheumatologic diseases in terms of impact on daily life and effectiveness of treatment. In this sense, Patient-Reported Outcomes (PROs) allow clinicians to better assess the health state of patients in terms of self-reported evaluation of quality-of-life and functional status without interpretation of the patient response by a healthcare professional. PROs regard the patient’s perception of symptoms, function and other aspects of daily life potentially disrupted by the disease (6). The assessment of disease status and the effectiveness of treatments from a patient’s point of view are essential aspects of managing PsA. Patient-reported outcomes measures (PROMs) are tools or instruments used to measure PROs. They are often patients’ self-completed questionnaires investigating health-related quality-of-life, symptoms and symptom burden, personal experience of care, psychological endpoints, and health-related behaviors such as Anxiety or Depression. According to different studies (7–9), PROM changes correspond to clinicians’ other objective measures of disease activity. PROMs were described as good predictors of long-term outcomes in different studies and are commonly used in clinical pharmacology trials as primary or secondary outcome measures. In fact, as a part of the OMERACT PsA Core Domain Set for PsA (10–16), PROMs are expected to be measured in all PsA RCTs and physician assessments of joints and skin. However, data on outpatients’ assessment using PROMs in real-world settings are scarce. They might be helpful in real-world clinical practice, outlining the relevant role of a specialized nurse to implement their use in clinical practice, improving the quality of care. The study aimed to assess PROs and the relationship between PROMs and the DAPSA score in consecutive outpatients affected by PsA.

## Materials and methods

A cross-sectional study was conducted from march 2018 to October 2020. PsA outpatients who fulfilled the ClASsification criteria for Psoriatic Arthritis (CASPAR), peripheral subset, aged ≥18 years, were consecutively enrolled at the rheumatology and dermatology outpatients’ clinic of the Department of Internal Medicine of the National Relevance and High Specialization Hospital Trust ARNAS Civico, Di Cristina, Benfratelli in Palermo (Italy). Each patient signed a written informed consent and was evaluated for socio-demographic data such as gender, age, smoking status, disease duration, work status, and therapy. PROMs data were collected according to the OMERACT PsA Core Domain Set (14). The Disease Activity in Psoriatic Arthritis 66/68-joint count (DAPSA 66/68) score was used to evaluate joint manifestations, and it indicates a remission condition (score 0–4) and a low (score 5–14); moderate (score 15–28) or high disease activity (score > 28). Fatigue was assessed with the FACIT-F (17); fatigue was classified in our sample based on three score classes (0–20), (20–40), and (>40). Depression was investigated using the Patient Health Questionnaire (PHQ-9). PHQ-9 scoring subdivided the sample into no depression (0–4), mild (5–9), moderate (10–14), moderate–severe (15–19) or severe (20–27) depression (18). Functional disability was assessed using the Health Assessment Questionnaire (HAQ) and scored as mild to moderate difficulty (0–1), moderate to severe disability (1–2), and severe to very severe disability (2–3) (19, 20). The Psoriatic Arthritis Impact of Disease (PsAID) was used to assess the impact of the disease on patients’ quality of life. According to PsAID, a score between 0 and 3 was considered a patient-acceptable status; a score between 4 and 10 indicated a patient-non-acceptable status, so a high impact of the disease on the patient’s life (21). The Visual Analog Scale (VAS) was used to report pain. Laboratory markers of inflammation, including C-reactive protein (CRP; mg/l), were collected. The treatment regimen was registered.

### Ethics

The study was conducted following the ethical standards laid down in the 1975/83 Helsinki Declaration and its later amendments, and informed consent was obtained from all participants included in the study. Ethics Committee Palermo2 approved the study with protocol number 231/CIVICO/2018.

### Statistics

Data were reported as percentages for categorical variables, and score values were represented as median (first quartile – third quartile). Statistical analysis was performed using the linear regression analysis to evaluate the relationship between DAPSA Score and all the included PROMs (HAQ, PsAID, FACIT-F and PHQ-9). We represented graphically our data by regression line with 95% confidence intervals and dots as data of each patient. Adjusted Rsquare was used to show the strength of association between variables. A two-tailed *p* < 0.05 was considered statistically significant. STATA [StataCorp.2021]. Stata Statistical Software: [Release 17, College Station, TX, United States: StataCorp LP] was used for database management and analysis.

## Results

From march 2018 to October 2020, 158 outpatients aged 55.2 years (53.3–57.1) affected by Psoriatic Arthritis (peripheral subset) were consecutively enrolled and evaluated according to CASPAR criteria. The demographic and clinical characteristics of the enrolled sample are shown in Table 1. Both genders were equally represented. The median years of illness of the sample was 10.6 (9.3–11.9). Very few of them had higher levels of education (9%), 24.7% were smokers, and only 5.7% consumed a regular amount of alcohol daily. Patients were currently treated with csDMARDs (78% methotrexate, 10% leflunomide, 7% sulfasalazine, 5% cyclosporine) or bDMARDs (69% anti-TNF- alfa, 1% anti IL-23.4% anti-IL12-23, 13% anti-IL 17, and 13% small molecules) and 74.5% of them were treated with combinations of both bDMARDs and csDMARDs. DAPSA score showed 10.8% of the sample with a remission of the disease and 65.8% of them with moderate to high disease activity.

**Table 1:** Demographic and clinical characteristics of our population sample.

N°	158
Female (%)	54.4
Age (years)^*^	55.5 (47.1–63.6)
Age men^*^	56.2 (47.2–63.6)
Age women^*^	55.4 (47.1–63.7)
Disease duration (years)^*^	10.6 (9.3–11.9)
Subjects without caregivers (%)	38.6
Education (%)	
Elementary school	15
Secondary school	45
High school	31
Degree	9
Family members^*^	3 (2–4)
Smokers (%)	24.7
Alcohol consumption (%)	5.7
CASPAR^;^ Criteria	5 (4–6)
DAPSA^§^	19.02 (9–33.1)
Facit Fatigue*	35 (20–43)
PHQ-9^¥^	7 (4–13)
HAQ^¶^	1 (0.25–1.75)
PSAID^$^	4 (2–6)
CRP (mg/l)*	1.47 (0.34–3.05)
66 Swollen Joint Count*	2 (0–6)
68 Tender Joint Count*	4 (0–13)
PASI^:^	1 (0–2)
csDMARDs^||^	41.6
Methotrexate (%)	78
Leflunomide (%)	10
Sulfasalazine (%)	7
Cyclosporine (%)	3
Hydroxychloroquine (%)	2
bDMARDs^**^	81.3
Anti TNF-Alpha (%)	69
Anti IL 17 (%)	13
Small Molecules (%)	13
Anti- IL 12–23 (%)	4
Anti IL-23 (%)	1
Combination of csDMARDs and bDMARDs	74.5

Population characteristics. ^*^Data are reported as median (interquartile range). Data are presented as n (%). CASPAR (ClASsification criteria for Psoriatic Arthritis); §DAPSA (Disease Activity in Psoriatic Arthritis); ¥PHQ-9 (Patient Health Questionnaire-9); ^¶^HAQ (Health Assessment Questionnaire); $PSAID (Psoriatic Arthritis Impact of Disease); PASI (Psoriasis Area Severity Index); ||csDMARD(Synthetic disease-modifying anti-rheumatic drugs); **bDMARD (Biologic Disease-modifying Anti-rheumatic Drug).

According to PROMs evaluation (Table 2), the FACIT-F showed our enrolled sample divided into three score classes: (0–20) 22.2%, (20–40) 46.8%, (>40) 31%. The first and the second score classes show 69% of the PsA population with high or moderate fatigue.

**Table 2:** Distribution into severity categories of DAPSA score and patients reported outcomes.

N°	158
DAPSA^*^	
Remission (%)	10.8
Low disease activity (%)	23.4
Moderate disease activity (%)	36.1
High disease activity (%)	29.7
Facit fatigue	
Score 0–20 (%)	22,2
Score 20–40 (%)	46,8
Score ≥ 40 (%)	31,0
PHQ-9 ^¥^	
No depression (%)	28
Mild depression (%)	32
Moderate depression (%)	18
Moderate–severe depression (%)	15
Severe depression (%)	7
HAQ^¶^	
Mild to moderate difficulty (%)	51
Moderate to severe disability (%)	31
Severe to very severe disability (%)	18
PSAID^$^	
Patient-acceptable status (%)	47.5
Patient-non acceptable status (%)	52.5

Patients reported outcomes in the enrolled sample. Data are presented as n (%). ^*^DAPSA (Disease Activity in Psoriatic Arthritis); ^¥^PHQ-9 Patient Health Questionnaire-9; ^¶^HAQ Health Assessment Questionnaire; ^$^PSAID Psoriatic Arthritis Impact of Disease; For categorization for DAPSA and PROMs, see the method section.

According to the PHQ-9 questionnaire, 40% of the subjects had moderate to very severe Depression. The median HAQ was 1 (0.25–1.75). Data analysis showed 51% of patients with normal-mild functional disability, 31% with moderate to severe disability, 18% with severe to very severe disability, and, according to the PsAID questionnaire, 52.5% of the patients had a high impact of the disease on life.

**Figure 1:**
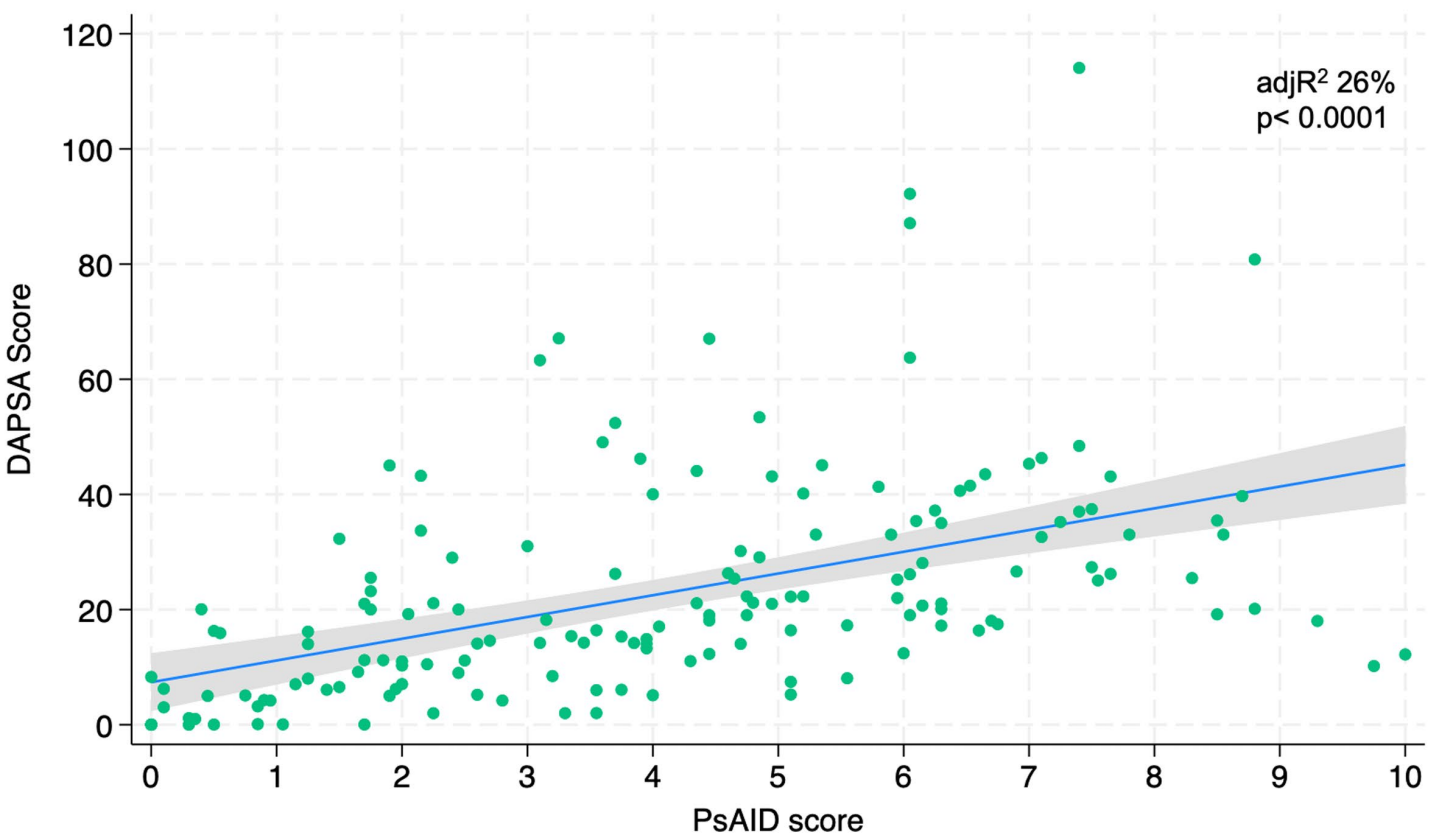
Linear relationships between DAPSA and PsAID.

**Figure 2:**
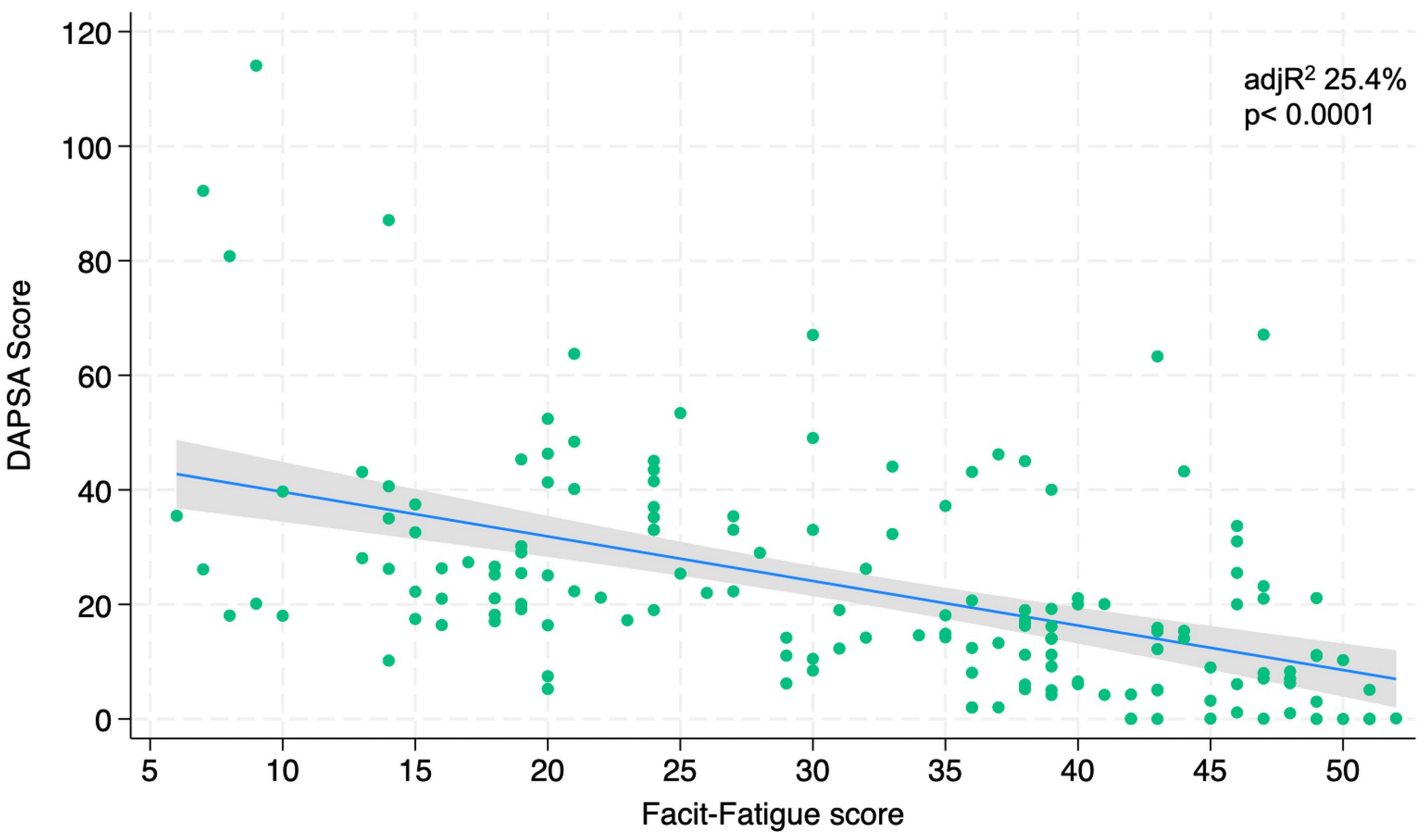
Linear relationships between DAPSA and Facit-F.

**Figure 3:**
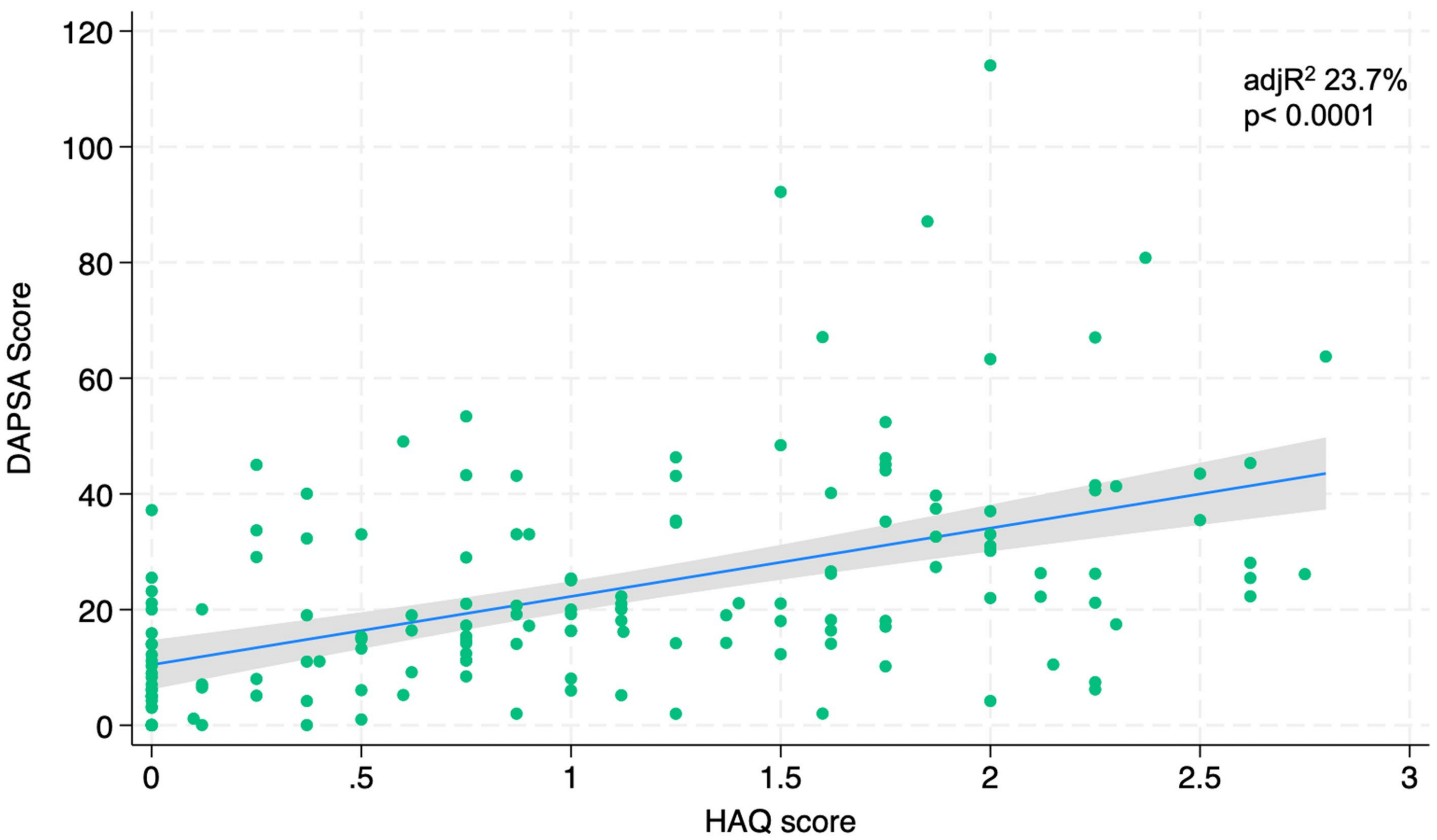
Linear relationships between DAPSA and HAQ.

**Figure 4:**
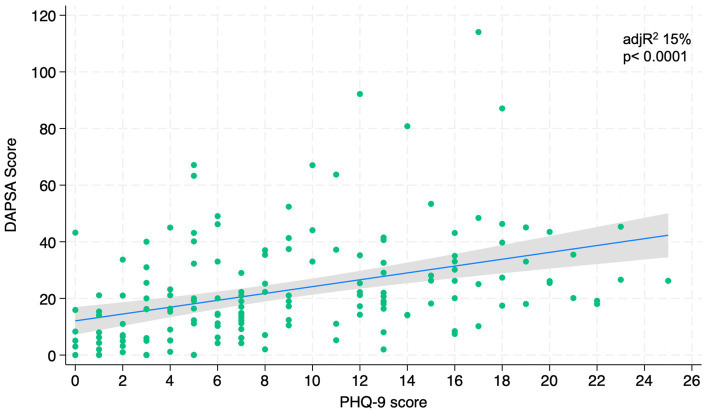
Linear relationships between DAPSA and PHQ-9.

Figures 1–4 represents linear relationships between variables, DAPSA score versus PsAID, Facit-F, HAQ, and PHQ-9, respectively. Adjusted R-Squared (adjR^2^) and relative value of p are reported in each figure as measure the strength of association between variables. Regression line with their 95% confidence intervals and dots as data from each patient were drown. The regression analysis highlighted a relationship between the DAPSA score and all the PROMs included in the study. The most significant analyzed relationships were between the DAPSA score and the PsAiD (adjR^2^ 26%, *p* < 0.0001) as shown in Figure 1, the DAPSA score and the FACIT-F (adjR^2^ 25.4%, *p* < 0.0001) as shown in Figure 2, and between DAPSA score and HAQ (adjR^2^ 23.7%, *p* < 0.0001) as shown in Figure 3. Relationship between DAPSA Score and PHQ-9 score is shown in Figure 4 (adjR^2^ 15%, *p* < 0.0001).

## Discussion

PsA is a heterogeneous, multidimensional, chronic inflammatory disease with a variable presentation that negatively impacts patients’ quality of life.

The impact of PsA is broad, covering different aspects of life, including functional and emotional aspects, but also domains such as fatigue and pain.

Over the last few years, greater importance has been given to the patient’s perception of his health status, especially in the rheumatology field, and the approach to outcome measures has changed profoundly. Rheumatologists evaluate the disease activity in PsA patients with the DAPSA score, a metric instrument. However, the patient’s perception of care is also essential to improving their quality of life, and nurses have a central role in this assessment.

Moving from a physician’s point of view to a patient-centered perspective, the introduction of the use of PROMs in the global evaluation of PsA patients is important, and the nurse is involved as the healthcare professional who can educate the patient in the self-assessment. Incorporating PROMs into the clinical assessment fosters a more collaborative approach between healthcare professionals and patients. By actively involving patients in the assessment process, physicians can empower them to take ownership of their healthcare and actively participate in decision-making regarding their treatment options.

Recently, the Group for Research and Assessment of Psoriasis and Psoriatic Arthritis (GRAPPA) treatment recommendations for PsA highlighted that a multidisciplinary and multispecialty approach is necessary to optimize PsA management and, according to that, the ideal clinical assessment might include PROMs next to metrics instruments validated for PsA such as the DAPSA score (22).

In order to globally standardize data, a core domain set of PROMs, including HAQ, PsAID, Facit-f, PHQ-9 and more, was endorsed as The OMERACT Core Domain Set for PsA, and its use in research is highly recommended (23).

Therefore, in patients affected by PsA, core domains (7) such as disease activity, function, and patient quality of life are crucial to assess patients globally, and the tools afore are described.

In the present study, we assessed consecutively outpatients affected by PsA in a real-world setting based on the Core Outcome Measurement Set for Psoriatic Arthritis (7, 13–15, 24, 25), including Facit-F, PsAID, PHQ-9 and HAQ scores. We evaluate the association of those PROMs with a disease activity measurement as the DAPSA score.

According to our study, the DAPSA score showed only 10% of the enrolled sample with remission of disease activity in accordance with the literature (26).

In different studies, fatigue was more frequently reported among patients with PsA than patients with cutaneous psoriasis alone and, according to that, more than half of our sample suffered from moderate to severe fatigue (27, 28).

Depression is a recognized but understudied comorbidity in patients with PsA, and according to the PHQ-9 questionnaire, 40% of the subjects had moderate to very severe depression. The prevalence of depression is significantly higher in this patient population than in the general population (29).

Our results showed that all the PROMs included in the analysis were associated with the DAPSA score and, in particular, significant associations were found with the PsAID, the FACIT-F, the HAQ, and the PHQ-9 in order to the strength of the association (adjR^2^ 26, 25.4, 23.7, 15%, respectively).

In 2016, Gudu et al. highlighted that fatigue in PsA was related to disease severity (30); in fact, our findings showed that PsA fatigue was significantly associated with DAPSA score in accordance with Lai et al. In particular, fatigue is a multifactor disease related to different conditions, so the authors need to include psychological, emotional, physical and quality-of-life measurements in the global assessment of a PsA patient (8).

We have also demonstrated that PSAID significantly correlates to the DAPSA score, that is a measure of disease activity. Due to this close relation between PSAID and DAPSA, PsAID might be recommended as a “patient-reported disease activity index.” However, it is known that the PSAID is not a disease activity index, but it is an excellent PRO to evaluate the effect of PsA on patients’ life as Di Carlo et al. showed in their study (31).

According to the literature and our results, the functional impairment assessed with the HAQ is also related to disease activity. In fact, in a study conducted on 440 patients enrolled in two different trials, participants in the different disease activity states according to the DAPSA score had different degrees of functional impairment, with a HAQ disability score twice as high in patients with high disease activity than in those with low disease activity (5).

Prior studies have examined the association between mood and disease activity in PsA (9), but not according to the PHQ-9 used in our study. Wong et al. state that depression and anxiety might reduce the probability of achieving a state of sustained minimal disease activity in patients with psoriatic arthritis (32). However, our results showed that worse mood was associated with worse DAPSA.

Our findings provide useful information for understanding that some patient-reported outcome measures are as important as disease activity indexes in assessing PsA patients. The current data highlighted the importance of assessing PsA globally and standardizing it to improve the quality of care for those patients. The strength of this study was the accuracy of the collected data; however, this is a single-center study, and the limitation can be overcome with a multicenter study.

In conclusion, patient-reported outcomes measures are essential tools for comprehensively evaluating the impact of PsA on patients. By incorporating PROMs into the clinical assessment, the interprofessional team can gain a deeper understanding of the patient’s experience, tailor treatment plans more effectively, and monitor treatment response more accurately. This patient-centered approach can lead to improved patient outcomes, increased satisfaction with care, and enhanced adherence to treatment recommendations. Furthermore, as demonstrated by our results, different PROMs are robust tools also to evaluate a patient’s clinical status as the DAPSA score. PROMs allow physicians and healthcare professionals to multidimensionally evaluate the patients to assess the impact of the disease on their lives and might be included in real-world settings, to evaluate patients better. Further research is needed to enroll more patients in a multicenter study.

## Data availability statement

The raw data supporting the conclusions of this article will be made available by the authors, without undue reservation.

## Ethics statement

The study was conducted following the ethical standards laid down in the 1975/83 Helsinki Declaration and its later amendments. Ethics Committee Palermo2 approved the study with protocol number 231/CIVICO/2018. Informed consent was obtained from all participants included in the study.

## Author contributions

ML: Conceptualization, Data curation, Investigation, Methodology, Project administration, Supervision, Validation, Visualization, Writing – original draft, Writing – review & editing. RM: Conceptualization, Investigation, Methodology, Project administration, Supervision, Validation, Writing – original draft, Writing – review & editing. GN: Data curation, Formal analysis, Software, Writing – review & editing. AG: Investigation, Methodology, Project administration, Writing – review & editing. IC: Investigation, Methodology, Project administration, Supervision, Writing – review & editing. SS: Methodology, Supervision, Writing – review & editing. CA: Conceptualization, Data curation, Methodology, Validation, Writing – original draft, Writing – review & editing. SC: Conceptualization, Data curation, Formal analysis, Investigation, Methodology, Project administration, Supervision, Validation, Writing – original draft, Writing – review & editing.
